# Review of Liquid Chromatography-Mass Spectrometry-Based Proteomic Analyses of Body Fluids to Diagnose Infectious Diseases

**DOI:** 10.3390/ijms23042187

**Published:** 2022-02-16

**Authors:** Hayoung Lee, Seung Il Kim

**Affiliations:** 1 Research Center for Bioconvergence Analysis, Korea Basic Science Institute (KBSI), Ochang 28119, Korea; lhy3221@kbsi.re.kr; 2 Bio-Analytical Science Division, University of Science and Technology (UST), Daejeon 34113, Korea

**Keywords:** bodily fluid, biomarker discovery, COVID-19, infectious disease, mass spectrometry, pathogen, proteomics

## Abstract

Rapid and precise diagnostic methods are required to control emerging infectious diseases effectively. Human body fluids are attractive clinical samples for discovering diagnostic targets because they reflect the clinical statuses of patients and most of them can be obtained with minimally invasive sampling processes. Body fluids are good reservoirs for infectious parasites, bacteria, and viruses. Therefore, recent clinical proteomics methods have focused on body fluids when aiming to discover human- or pathogen-originated diagnostic markers. Cutting-edge liquid chromatography–mass spectrometry (LC-MS)-based proteomics has been applied in this regard; it is considered one of the most sensitive and specific proteomics approaches. Here, the clinical characteristics of each body fluid, recent tandem mass spectroscopy (MS/MS) data-acquisition methods, and applications of body fluids for proteomics regarding infectious diseases (including the coronavirus disease of 2019 [COVID-19]), are summarized and discussed.

## 1. Introduction

Infectious diseases are major threats to global health, as evidenced by the current coronavirus disease of 2019 (COVID-19) pandemic. Even though many treatments for infectious diseases are available, they remain one of the leading causes of death in the world [1,2,3,4]. Therefore, it is crucial to better understand the physiology of pathogens (bacteria, viruses, fungi, and parasites) at the molecular level [5]. The first step when studying bacterial pathogens involves identifying molecular features that contribute to pathogenicity. These features can be potential therapeutic targets, and their inhibition can eradicate or counteract bacterial infections.

Proteomics is a useful tool for studying infectious diseases because it can provide large-scale protein information involved in the pathogenesis, infection mechanisms, and pathological symptoms of hosts. Modern proteomic methods have evolved from gel-based techniques to gel-free mass spectrometry (MS) approaches known as “shotgun” proteomics. Shotgun proteomics utilizes nano-high precision liquid chromatography (HPLC) systems coupled to high-resolution mass spectrometers; it has revolutionized the proteomic research field by allowing large-scale protein characterization with high throughput [6]. For the discovery of biomarker candidates or pathogen detection in the infected host, MS-based proteomics have several advantages. MS-based proteomics have high performance regarding its detection limits (<1 nM), repeatability, and reproducibility, in comparison to immunoassays. The advantage of this technology is that it can be conducted in a multiplexed manner, without the use of antibodies or comparable binders [7]. In addition, MS-based analyses are now generally integrated into many hospital laboratories for the routine identification of bacterial pathogens in clinical samples, as well as for antibiotic resistance testing. Proteomic studies related to infectious diseases can be categorized into three groups according to target samples as follows: (1) pure-cultured pathogen, (2) infected host proteome, and (3) pathogens in the infected host.

(1) Pure-cultured pathogens: the proteomic analysis of pathogens grown under pure culture conditions has several advantages regarding the characterization of pathogens. First, this approach provides accurate translational information of each gene at the genome-wide level. Second, it is easy to control the cell culture conditions of pathogens and acquire their proteomic responses. However, the pure-culture conditions of pathogens are different from real infection conditions. In general, host systems infected with pathogens provide more severe and diverse culture conditions. Additionally, many pathogens related to human diseases cannot be cultured in laboratory environments [8].
(2) Infected host proteome: the host proteome infected with pathogens is another important target for proteomic analysis. This approach can provide valuable information on host–pathogen interactions, the infection mechanisms of pathogens (pathogenic bacteria or viruses), and the pathological symptoms of hosts. The study of the interactions between microbial pathogens and their hosts is called “infectomics”; it constitutes a growing area of interest in proteomics. Infection sites within a host are also diverse, including the respiratory system, digestive system, nerve systems, skin, and body fluid. Therefore, many clinical samples are available. However, though host-derived biomarkers are useful for monitoring disease status, they are limited for discerning between similar diseases [9].
(3) Pathogens in the infected host: detecting pathogens (pathogenic bacteria or viruses) from an infected host is the most direct method for the diagnosis, prognosis, treatment, and clinical characterization of infectious diseases. Body fluids can be useful samples for this analysis.

**Figure 1:**
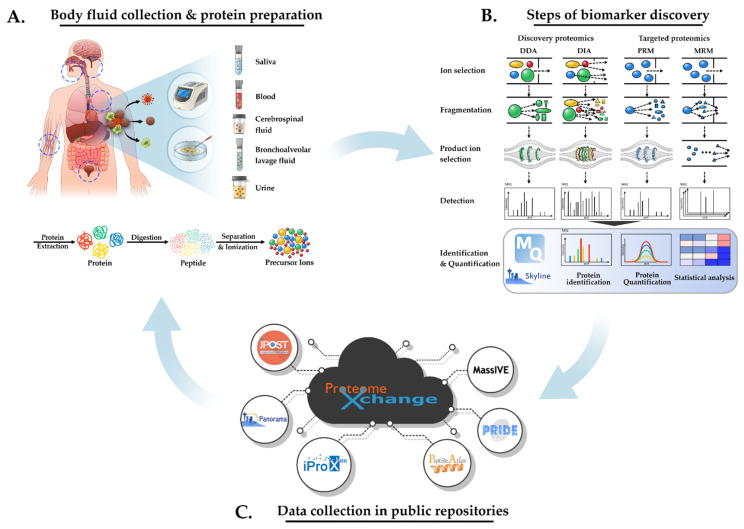
Schematic depictions of liquid chromatography-mass spectrometry (LC-MS) to discover biomarkers for infectious diseases. These workflows are designed to discover pathogen-originated biomarkers. (**A**) Body fluid collection and protein preparation. Body fluids are useful for diagnosing infectious diseases. The body fluids of patients with infectious diseases are screened using polymerase chain reaction (PCR) or culture tests and then collected. Proteins are extracted from body fluids and enzymatically digested into tryptic peptides. The resulting peptides are applied to LC/MS for separation and ionization. (**B**) The steps of biomarker discovery using various acquisition methods. Protein identification, quantification, and statistical analysis methods are used to identify useful biomarkers. Mass spectroscopy (MS) analysis is categorized by discovery proteomics (data-dependent acquisition [DDA] and data-independent acquisition [DIA]) and targeted proteomics (parallel reaction monitoring [PRM] and multiple reaction monitoring [MRM]). The principles of each mass technology are described in detail in Section 2.2. The advantages and disadvantages of each approach are summarized in Table 3. Acquired fragmented spectra are translated into peptide sequences and then inferred to identify proteins using proteomics software such as MaxQuant or Skyline. The intensities or peak areas of the acquired peptides are used for comparative analysis of the corresponding proteins between clinical samples. As a next step, various statistical analyses of MS data can help to discover potential biomarkers indicative of specific infectious diseases. (**C**) Data collection in public repositories for further applications. Many MS data produced by previous studies can be deposited in public repositories. These data should be further curated to be housed in an open database, which can be used for discovery or validation studies.

Thus, MS-based proteomics are expected to be used in the future as a tool to rapidly identify pathogens from human biological specimens. Figure 1 shows an overview of LC-MS based proteomics to discover biomarkers for infectious diseases. However, to date, the direct detection of pathogens from the host is still difficult to routinely implement, owing to several technical hurdles. At this point, this review is about the detection of pathogens in the body fluids of an infected host. At first, it covers the characteristics of each body fluid as samples for clinical proteomics, in Section 2 (Figure 1A). It also briefly introduces current proteomics approaches (data-dependent acquisition/data-independent acquisition (DDA/DIA), and targeted proteomics) that have been applied to identify the pathogen in the infected host, in addition to reviewing a coronavirus disease-19 (COVID-19) case to showcase the most up-to-date technology applied, in Section 3 (Figure 1B). Afterwards, future challenges are discussed in Section 4 (Figure 1C).

## 2. Using Body Fluids for the Proteomic Analysis of Infectious Diseases

### 2.1. Body Fluids as Valuable Clinical Samples for Proteomic Analysis

As described in the introduction, proteomic targets for infectious diseases can be categorized into three groups. Among them, two targets (host-infected with pathogens and pathogens in the infected host) are important for screening clinically available biomarkers for diagnosis or treatment [10]. Body fluids can be used as the main source of these targets [5,11]. Body fluids are defined as “liquids within the human body”; they can be classified as being either systematic or proximal (Figure 1A). Systematic fluids represent the host’s overall physiological state. However, proximal fluids are limited to specific tissue, but represent the status of adjacent tissue.

The advantages of body fluids include the fact that they are easier to collect with less invasive methods, with less demanding sample preparation methods than those with the tissue before proteomic analysis [12]. Several body fluids can be analyzed, but we chose five commonly studied body fluids. The characteristics of the representative body fluids are summarized in Table 1.

Blood is the most popular body fluid for host proteomic analysis because it represents the proteome of the whole organism, as it contacts every tissue in the body. It also contains many hidden or unknown proteins, such as cytokines, hormones, and antibodies, thus providing insights on the clinical or physiological conditions of the host [23]. Both these characteristics are valuable aspects that other body fluids do not have. Therefore, blood is a useful clinical sample to guide the treatment of various diseases. Serum or plasma is the blood fraction used for this purpose. However, fewer than 15 of the most abundant but least informative proteins account for more than 90% of the total protein components of these fractions. The high complexity of protein contents and additional modifications make the analysis more complicated [24]. Strategies for removing these abundant proteins without losing informative low-copy proteins are essential to ensure the effective screening of most indicative biomarkers. There are still technical challenges in identifying low-abundance proteins in the blood.

Bronchoalveolar lavage fluid (BALF) is a biofluid obtained using fiber-optic bronchoscopy. This fluid reflects the protein composition of the pulmonary airway. Therefore, proteomic analysis of the BALF can provide information on airway diseases. BALF can be a more sensitive clinical sample than nasal swabs regarding respiratory molecular diagnostics, but its sampling method is ineffective and invasive and requires considerable time and costs. The BALF proteome is dominated by plasma-derived proteins such as albumin and immunoglobulins (65–80%), which makes its analysis difficult [25]. However, BALF can be a valuable clinical sample for the screening of lung-specific disease biomarkers [14].

Cerebrospinal fluid (CSF) is a body fluid that surrounds the ventricular system of the central nervous system, spinal cord, and brain. CSF transports waste products and nutrients, mediating molecular exchange with blood plasma. As it connects with the blood system, most of its protein contents are similar, but the protein concentration is lower than that of plasma [15,26]. CSF also acts as a mechanical support for the spinal cord and brain. It is considered to be an ideal clinical sample for the detection of neurological disorders or diseases, such as multiple sclerosis, meningitis, and spinal cord injuries. However, the disadvantage of CSF is its associated invasive sample collection method, which requires lumbar puncture. Blood contamination and the presence of blood plasma proteins in the CSF are further disadvantages regarding the identification of biomarkers by CSF proteomic analysis [15].

Urine is a liquid secreted by the kidneys; it is the result of glomerular filtration of the plasma to eliminate waste products, such as urea and metabolites. It also includes proteins secreted from the urinary tract and renal tubular epithelial cells. Therefore, urine covers related diseases of the blood, kidneys, bladder, and urinary tract. One advantage of using urine as a clinical sample is that it can be collected in large quantities in a non-invasive manner. It is also possible to collect urine samples repeatedly, which aids in time-resolved studies. Furthermore, urine has a 1000-fold lower proteome complexity (0.08 mg·mL^−1^) than serum or plasma [27]. Finally, proteins or peptides in urine are stable because the urine proteome remains in the bladder for a considerable time before excretion, after the proteolytic process mediated by endogenous proteases. These characteristics make urine a convenient source for discovering biomarkers. To date, the major targets in urine proteomics have involved the screening of biomarkers of renal and urogenital dysfunctions [28], but it has also been applied to other infectious diseases described later [29,30,31,32,33]. Saliva is a fluid secreted by the salivary glands and gingival crevice [34]. It contains more than 1000 proteins that originate from glands and plasma [16]. Cystatins, α-amylase, mucin, albumin, globulins, and serotransferrin are the major proteins [35]. As the protein complexity of saliva is relatively low, it is preferred for biomarker screening by proteomic analysis. Like urine, saliva has the advantage of easy and non-invasive sample collection. Periodontal diseases, such as periodontitis, oral cancer, autoimmune disease, and diabetes mellitus, are popular targets of saliva proteomics [34,35,36].

The standard operating procedures (SOPs) of authorized institutions should be considered to obtain reproducible results (Table 1). This will be helpful to minimize pre-analytical variables while ensuring quality and uniformity among samples. There are some excellent reviews summarizing detailed information on SOPs [14,19,20,21,22]. Although each body fluid has unique characteristics, several steps for obtaining high-quality results are similar. The commonly considered factors related to protein sample preparation are summarized in Table 2. 

For example, sample fractionation or depletion is commonly required before tandem mass spectroscopy (MS/MS) analysis regardless of the type of body fluids because of the proteome complexity or the presence of high abundance proteins in body fluids [12,27]. In the case of blood, the dynamic ranges differ by a factor of 10^10^ between serum albumin (35–50 mg/mL in normal conditions), the most abundant protein, and cytokines (low pg/mL range). Unfortunately, this needs to be considered because immunodepletion can also co-deplete low-abundance proteins. Several studies have shown limited success in depleting high-abundance proteins and enriching low-abundance proteins [37,38,39,40]. Alternative methods to simplify sample complexity, such as fractionation, have been applied optionally, which increases the number of LC-MS/MS runs. The advantages and disadvantages of each fractionation method have been described well previously [18,45,47].

The solubility of target proteins needs to be considered because avoiding sample loss owing to precipitation or aggregation and efficient digestion into peptides by proteases are prerequisites for MS-based bottom-up analysis [48]. Several MS-compatible reagents are commercially available to enhance the solubility of proteins or enzymatic digestions. Waas and colleagues evaluated the efficiency of eight commercially available reagents based on the number of peptides and proteins identified, total protein sequence coverage, and digestion specificity in various conditions [41].

The digestion methods are the major bottle necks for large-scale projects. They are also the source of experimental variability and need to be performed as consistently as possible. To develop an optimal process, an extensive set of protocols have been developed which are divided into two categories: in-solution process and cleanup methods [49]. In-solution processing methods include in-solution digestion [50] and integrated StageTip workflow [51]. Those have simple protocols and can be adapted to high-throughput processes, but the presence of reagents can hinder downstream processes. Cleanup methods include in-gel methods, filter-aided sample preparation [52], suspension trapping [53], single-pot, solid-phase-enhance sample-preparation (SP3) [49], precipitation, and other affinity-based methods. Those methods provide high-quality results but require time consuming and laborious processes. Recently, Müller and colleagues proposed fully automated high-throughput and streamlined workflows for clinical samples using SP3 methods, which can deal with 96 samples in 3.5 h [44]. A detailed comparison of these methods is beyond the scope of this paper. However, several studies have described the differences among such methods [18,42,43,46,49,53].

Over 500,000 peptides can be derived from body fluid proteins per sample. The hundreds of co-eluting peptides were ionized and analyzed together. The high dynamic range and difference in ionization efficiency of the co-eluting peptides can affect MS/MS analysis [54]. Peptide prefractionation or enrichment is one way to solve this problem. Those procedures are usually achieved by chromatographic and electrophoretic fractionation to reduce the number of co-eluents. Strong-cation exchange coupled with reverse phase (RP), high-pH RP coupled with RP, or hydrophilic interaction and RP chromatography are commonly applied for peptide prefractionation [18,46]. An interesting reference paper by Wasinger and colleagues provides more detail of the separation of peptides using various one- or multidimensional methods for LC-MS analysis [45].

### 2.2. Increase in Applications of Body Fluids for Proteomics of Infectious Diseases

**Figure 2:**
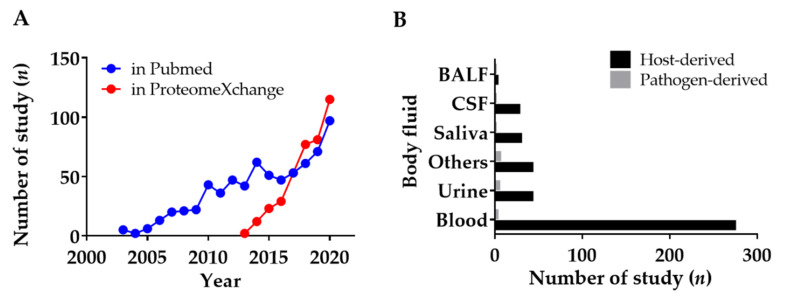
Current statistics of studies on body fluid proteomics for infectious diseases. (**A**) Gradual increases in research papers and deposited datasets on body fluid proteomics for infectious diseases. These results were based on PubMed and ProteomeXchage searches conducted in 2020 for the past 20 years (2002–2020). (**B**) Utilization of various body fluids for diagnosing infectious diseases divided based on the targets. These results were based on ProteomeXchage searches. The statistics showed that body fluid proteomics is an emerging proteomics field for pathogen-originated biomarker discovery.

With the spread of COVID-19, scientists and clinicians are now paying more attention to the rapid and effective diagnosis of infectious diseases than ever before. Body fluid proteomics has been considered an emerging technology to identify novel biomarkers. For this review of body fluid proteomics, more than 630 research papers related to body fluid proteomics were identified in PubMed using the following words: ((Biomarker) AND (Proteomics)) AND (Infectious disease OR Emerging disease) AND ((Plasm*) OR (Seru*) OR (bloo*) OR (Urin*) OR (Cerebro*) OR (Bronchoalveolar*) OR (Body fluid) OR (Liquid biopsy)) NOT (Cancer). Additionally, 422 deposited datasets related to infectious diseases were also found in the data repository proteomeXchange, which is a data repository for proteomics studies [55,56]. Continuous increases in research papers and deposited datasets related to body fluid proteomics for infectious diseases can thus be seen (Figure 2A). The deposited datasets were manually curated according to the type of body fluid and the type of targets in Figure 2B. Unexpectedly, only a few studies were discovered that targeted pathogen-derived proteins, which are major interests of this review. Most proteomic studies have focused on infected human or host proteomics.

## 3. Application of LC-MS Proteomic Analysis for Identification of Pathogens Using Body Fluids Associated with Infectious Diseases

MS analysis can be divided into three methods according to the acquisition method: DDA, DIA, and targeted-mass spectrometry (parallel reaction monitoring [PRM] and multiple reaction monitoring [MRM]). The characteristics of the acquisition methods are summarized in Table 3. Figure 1B also shows the fundamental concepts that each acquisition method. In this section, the characteristics of each method are briefly described, focusing on body fluid proteomics studies of infectious diseases that apply these MS methods for biomarker discovery, as summarized in Table 4.

### 3.1. Application of DDA for Proteomics of Infectious Diseases

LC-MS-based proteomics has evolved into two analytical methods: (1) discovery proteomics and (2) targeted proteomics [79]. The DDA, so-called “shotgun proteomics”, is a suitable method for discovery studies because it allows the comprehensive identification of bacterial proteins. In traditional DDA, protein samples are tryptic digested, following which the peptide mixtures are fractionated and analyzed by LC-MS/MS. The most abundant precursor ions in a given spectrum are then selected and fragmented into MS/MS for further analysis (Figure 1B) [80]. Various protein identification programs have been developed [64,81]. The most common approach for protein identification is the sequence database matching algorithm, in which real spectra obtained from MS/MS analysis are comparatively analyzed with in silico spectra derived from peptide sequences from a reference database. Therefore, using the correct high-quality searching algorithms and reference databases is essential for determining the quality of search results when using DDA. The high accessibility and wide coverage of DDA have made it the most widely used method (Table 3). However, stochastic sampling is the main limitation of DDA; it complicates the identification of low-abundance proteins in complex samples and, in some cases, low-abundance proteins are frequently ignored [82]. Owing to this problem, data are plagued with numerous missing values, therefore requiring imputation and resulting in the loss of statistical power when the sample size is increased. The depletion of abundant proteins or the fractionation of protein mixtures has commonly been applied to overcome this technical limitation [83]. Label-based protein quantification methods, such as tandem mass tags and isobaric tags for relative and absolute quantitation, are also routinely applied to the comparative quantitative analysis of the infected host proteome [84,85]. Optimizing MS/MS measurement conditions in LC/MS is also considered to be an important factor in expanding the usefulness of DDA [86,87].

There have been several important studies regarding the discovery of proteins of pathogen origin from clinical samples of infectious diseases. Kashino and colleagues applied the DDA approach to urine proteomics in patients with pulmonary tuberculosis (TB) [29]. The urine samples were prepared by filtration through a 5 kDa molecular weight cut-off (MWCO) filter. They found four proteins (MT_1721, MT_1694, MT_3444, and MT_2462) of Mycobacterium tuberculosis (*Mtb*) from nine patients with culture-confirmed pulmonary TB. Further validation of these proteins was performed by western blotting using anti-sera from patients with TB. Pollock and colleagues selected one candidate protein (MT_1721 or Rv1681) for further study [30]. This protein was confirmed by LC-MS analysis, and the full length of the target protein was validated using immunoaffinity precipitation MS analysis [30]. However, because of the low sensitivity of the DDA approach used in this study, the detection rate of the target protein (MT_1721) in the group of patients was not significantly high (less than 20%). The antibody of the target protein (MT_1721) was subjected to an enzyme-linked immunosorbent assay (ELISA) using approximately 100 clinical samples. ELISAs for the target proteins showed a detection rate of <50%. However, the authors confirmed the complete absence of urine reactivity in the negative controls. Young and colleagues also performed urine proteomics to discover TB-specific biomarkers using clinical samples obtained from patients with TB (*n* = 63) [31]. TB patients were categorized as having definite TB (*n* = 21), presumed latent TB (*n* = 24), or presumed non-TB (*n* = 18). The clinical samples were pretreated by filtration (50 kDa MWCO filter) and concentration (3 kDa MWCO filter) to deplete highly abundant proteins before proteomic analysis. Using the DDA approach, the authors discovered 16 proteins originating from *Mtb*. Additionally, 27 human proteins were selectively identified in patients with active pulmonary TB.

However, although the previously described body fluid proteomics studies succeeded in identifying bacterial-derived markers, in many cases researchers failed to identify bacterial proteins because of intrinsic limitations, low quantity target proteins relative to the host proteins, and/or the absence of target proteins in existing databases, as mentioned above [65]. Spectral library searching is an alternative method for overcoming sensitivity-related limitations [81]. This is described in more detail in the next section. In brief, this technique is typically more sensitive and faster than the sequence database searching approach because it directly matches the spectra of peptide ions to spectra contained in libraries [88,89]. Hentschker and colleagues reported improved and faster results based on the proteome and phosphoproteome of pneumococci [90]. They applied a spectral library instead of a sequence database to identify more unidentified bacterial proteins. The spectral library was derived from MS/MS analysis of the culture cells; it was validated using synthetic peptides. They identified 76% of the theoretical proteome and 128 phosphorylated proteins in Streptococcus pneumoniae. This method is expected to be useful for body fluid proteomics.

### 3.2. Application of DIA for Proteomics of Infectious Diseases

Following its introduction in 2004, DIA has become a new strategy for systemically analyzing complex protein mixtures [91]. Unlike DDA, in DIA all ions present in a certain range of the m/z window are co-fragmented and collectively analyzed (Figure 1B). The DIA approach makes it possible to expand the profiles of proteomes and accurately quantify targeted proteins. This method can result in better experimental reproducibility than DDA methods [60,92,93,94]. DIA has the merits of both DDA and targeted approaches (selected reaction monitoring [SRM]/MRM and PRM). Therefore, it has become a popular technology in proteomics research [95]. However, it is still unable to overcome the depth of proteome coverage in DDA and the accuracy of MRM or PRM in measuring very low-abundance proteins (Table 3). High-resolution MS/MS acquisition at fast scan speeds is required for DIA-MS experiments. The most widely used hybrid instruments, QExactive and QE plus, are believed to have sufficient performance for DIA analysis. Although DIA is an extremely powerful method, it is more complex than DDA because of the difficulties of MS/MS spectral data analysis. Previously used peptide identification algorithms are not appropriate for DIA because of the complexity of the MS/MS spectrum of DIA [58,81]. In order to deconvolute complex spectra, spectral libraries are essential as reference databases. In general, spectral libraries contain intensity and peak information of non-canonical fragment ions generated by multiple DDA analyses of target samples [59]. Unfortunately, standardized pipelines have not yet been established [58,64]. The contents necessary for the practical application of DIA have been described in more detail in recent review papers [94,96].

Roux-Dalvai and colleagues conducted urine proteomics using DIA analysis and machine learning to identify pathogens in the urinary tract. In the first step, spectral libraries containing 31,096 peptides from 15 pathogen colonies were obtained. Then, the authors prepared 12 artificial urine sample replicates spiked with 15 bacterial species to verify the spectral library. As a result, 4319 peptides were obtained as detected spectra. To select informative features among them, machine learning was used to identify peptide signatures; 82 peptides were selected. Further validation of the selected peptides was conducted using PRM. They successfully predicted the predominant bacteria in clinical samples (*n* = 27) [33]. DIA has been applied for the proteome analysis of infectious diseases by targeting host proteins; it has also been applied for the rapid diagnosis of identified pathogens [97].

### 3.3. Application of Targeted-MS for Proteomics of Infectious Diseases

DDA has been routinely used to discover biomarkers from clinical samples, with further validation being achieved through rigorous statistical methods. This validation process requires accurate, reproducible, and highly robust methods for quantifying candidate biomarkers. However, the abovementioned major limitations of DDA, related to irreproducibility and imprecision, result from stochastic problems. Targeted proteomics, meanwhile, have been devised for the precise quantitative analysis of specific proteins or protein complexes. Representative targeted proteomics include SRM, MRM, and PRM [98,99]. SRM/MRM technology eliminates most non-targeted detection methods, which can reduce the noise signal and improve the detection sensitivity. In general, a triple quadrupole instrument is used for these technologies. Monitoring specific transition windows (a small range of m/z values of precursor/fragment ion pairs; Figure 1B) results in increased selectivity and sensitivity compared to those with DDA and DIA approaches. It is known that targeted methods are at least 5–10 times more sensitive than DDA when analyzing whole-cell lysates [92,100] (Table 3). However, the bottleneck in the development of SRM/MRM-based assays is the complicated procedure of the optimization process [101,102,103,104]. For example, it is important to choose the prototypic peptides, which are the unique peptides that empirically have a high chance of being observed.

PRM technology has been optimized based on quadrupole-orbitrap instruments to deliver an improved version of targeted proteomics. Unlike SRM/MRM, PRM involves the acquisition of full MS/MS scans of product ions in orbitrap, rather than selected fragment ions from predefined precursor ions. Therefore, this technology is more convenient because it does not require the selection and optimization of fragment ions. It can also be used for qualitative purposes, as in DDA approaches, to avoid false positives. In summary, this technique provides simplified and robust workflows but requires time-consuming optimization steps. Therefore, it is not suitable for discovery-based applications but is very useful for validation applications targeting low-abundance proteins present in body fluids [105]. Targeted-MS based diagnosis has inherent strength compared to immunoassays in that it can perform the analysis in a multiplexed manner with high selectivity and sensitivity, without an antibody, at a low cost if the lab has appropriate instruments and has developed the assay [7,106].

Several studies have successfully employed targeted proteomics to quantify biomarkers exposed in body fluids for infectious diseases. Kruh-Garcia and colleagues first developed an MRM assay for the antigen 85 complex (Ag85) mycobacterial proteins that are potential diagnostic biomarkers for TB. They compared the amount of the Ag85 complex (represented by Ag85A, Ag85B, and Ag85C proteins), in the secretome of various clades of *Mtb*, revealing precise discrimination among those highly homologous proteins [67]. In a further study, they expanded their proteomic results in the secretome to include TB patient serum [68]. They identified 250 targeted peptides using DDA proteomics of *Mtb*-infected macrophages and a mouse model. After a thorough optimization process aided by in silico analysis, they selected 76 peptides as target peptides, representing 33 mycobacterial proteins (including Ag85). Then, they performed an MRM assay, using serum exosomes from TB patients as clinical samples. As a result, for the first time, they suggested 20 mycobacterial proteins present in the serum exosomes of TB patients as potential biomarkers (*n* = 41). The same research team developed refined MRM assays using isotope-labeled peptide standards [69]; these assays can detect mycobacterial proteins in serum exosomes in the attomolar to femtomolar range.

Karlsson and colleagues successfully selected species-unique peptides of the Mitis group of the genus *Streptococcus*, using proteogenomic analysis. They characterized and identified more than 200 unique peptides from cell lysates of cultured cells using DDA proteomics [70]. They then expanded their platform to discover peptide biomarkers of representative respiratory tract pathogens, including *S. pneumoniae, Haemophilus influenzae, Moraxella catarrhalis*, and *Staphylococcus aureus*. For the discovery phase, representative genetic variations were preselected as MS-inclusion lists and validated in bacterial culture proteomics. Finally, the targeted peptides of each of the four pathogens were confirmed in 218 clinical samples [71].

Wang and colleagues used a similar approach to identify five gram-negative pathogens in the BALF, including *Acinetobacter baumannii, M. catarrhalis, Pseudomonas aeruginosa, Stenotrophomonas maltophilia*, and *Klebsiella pneumoniae* [72]. Bardet and colleagues, meanwhile, developed an SRM-based method to rapidly and reliably identify pathogens using endotracheal aspirate samples of ventilator-associated pneumonia (VAP) [73]. Based on the high ionization yields of the unique peptides confirmed in DDA experiments, 97 species-specific peptides from the six most frequent bacterial species (*A. baumannii, Escherichia coli, H. influenzae, Pseudomonas aeruginosa, S. aureus*, and *S. pneumoniae*) responsible for VAP were selected and monitored using the developed SRM assay.

### 3.4. Application of LC-MS/MS for COVID-19 Diagnosis

The current COVID-19 pandemic, which is caused by severe acute respiratory syndrome coronavirus 2 (SARS-CoV-2), has justified the need for the development of diagnostic technology for infectious diseases. Molecular diagnostics such as polymerase chain reaction (PCR) have to date been used as the gold standard for the detection of SARS-CoV-2. However, novel alternative approaches have been introduced. Proteomics researchers have introduced novel LC-MS/MS-based diagnostic approaches for COVID-19 (Table 4).

Gouviea and colleagues first reported 101 tryptic peptides derived from six viral proteins identified from SARS-CoV-2-infected Vero E6 cells, using DDA analysis [74]. Through further curation, 14 peptides from nucleocapsid phosphoprotein (N protein), spike protein (S protein), and membrane glycoprotein (M protein) of the virus were recommended for further targeted MS. In a subsequent study, they proposed a time-efficient diagnostic method for COVID-19 clinical samples using LC-MS/MS as alternative methodologies to PCR or immunodiagnostic assays. They applied artificial nasopharyngeal swabs to evaluate 14 peptides. Among these 14 peptides, two peptides of the N protein were selected as attractive candidates [75]. Interestingly, the same target peptides were confirmed by independent groups using the PRM method [107,108]. However, neither approach could overcome the problems of the low detection rate (approximately 20% of the PCR assay) and low throughput analysis (20 min per sample). Thus, further investigations should aim to improve practical usage. Singh and colleagues also reported MRM assays using two other peptides derived from the S protein and replicase polyprotein, achieving significant results of 100% specificity and 90.5% sensitivity in a 2 min gradient run (*n* = 103) [76]. However, MRM measurements are limited by their low resolution, which makes it impossible to verify the peptide spectrum itself.

Cazares and colleagues reported a PRM assay for the detection of viral proteins in virus-spiked mucus samples and found that the limit of detection (LOD) and limit of quantitation (LOQ) were approximately 200 and 390 attomoles, respectively [109]. These values indicated that the assay could detect approximately 2 × 10^5^ viral particles/mL in a sample, showing comparable performance to the RT-PCR method.

Fully automated sample preparation and sample-cleanup methods with high-resolution MS seem to overcome these problems. Cardozo and colleagues developed a fully automated magnetic-based sample preparation method for nasopharyngeal and oropharyngeal swabs that could be completed within 4 h using a robotic liquid handler. Turbulent flow chromatography coupled with tandem mass spectrometry (TFC-MS/MS) can provide an efficient online sample cleanup method. This workflow can analyze four samples in a row within 10 min (in other words, more than 500 samples per day). The authors evaluated the target peptides of the SARS-CoV2 N protein qualitatively and quantitatively using PRM methods. The LOD and LOQ were reported to be 2–3 and 4–6 ng/mL, respectively. Compared to an RT-PCR-validated cohort, this workflow could detect up to 84% of the positive cases with a specificity of up to 97% (*n* = 985) [77]. Renuse and colleagues introduced automated immunoaffinity-based sampling combined with targeted high field asymmetric waveform ion mobility spectrometry (FAIMS) [78]. Acquired PRM data were used to model an “ensemble” machine learning-based classification method. This method obtained high-quality results, delivering 98% (86/88) sensitivity and 100% (88/88) specificity [78].

Rajczewski and colleagues thoroughly evaluated 636 viral peptides identified in datasets using Galaxy-based workflows [110]. Galaxy is a web-based platform that provides reproducible computational research and numerous bioinformatics tools. Using in vitro and clinical source datasets deposited in the public repository proteomeXchange, they selected four peptides derived from N and M proteins. These peptides were consistently detected across all datasets used in the study and were proposed as potential diagnostic biomarkers.

Additional studies from nasopharyngeal swabs, gargle solutions, or other human samples have also been published [109,111,112,113,114]. However, the results are limited, except for those of nasopharyngeal swabs, compared with the results of a PCR-based study [115,116]. During the initial phase, Ihling and colleagues reported PRM-based identification of N protein from patient gargle solutions [114]. Recently, Kipping and colleagues proposed an improved sample preparation protocol and developed MRM methods using a synthetic peptide library to target the N protein from gargle solutions and saliva [117]. Based on these results, LC-MS-based diagnostics seem to be in the beginning stage, except for the use of nasopharyngeal swabs. The SARS-CoV2 peptides that have been introduced as potential biomarkers in recent studies have been summarized in two previous review papers [118,119].

## 4. Concluding Remarks and Future Outlook

This review summarizes LC-MS-based proteomics for discovering biomarkers of infectious diseases, using various body fluids. Body fluid proteomics is an attractive method for monitoring patient status. The direct detection of pathogen-derived proteins or peptides from body fluids could also prove to be an optimal tool for identifying infectious diseases. However, studies into infectious diseases focusing on body fluid proteomics have not been actively performed due to technical difficulties. The first part of this review introduces the characteristics of the representative body fluids. Recent improvements in sample preparation methods have increased the coverage of proteome discovery. The second part describes the characteristics of representative MS acquisition methods such as DDA, DIA, MRM, and PRM. The application of body fluid proteomics to infectious diseases is also introduced here. Given the importance of the COVID-19 pandemic, recent results of COVID-19 studies using body fluids are also summarized here. Researchers have applied cutting-edge sample preparation methods and proteomic technologies to discover biomarkers and have reported improved results. For example, compared to the MRM assay developed by Kruh-Garcia in 2014 [68], which can process one sample per day, the automatic workflow developed by Cardozo in 2020 can handle 500 samples per day without laborious work [77]. In these studies, micro-flow LC with a short separation time was applied. This is contrary to the mainstream method of using nano-flow LC with a long separation time to obtain very high sensitivity. This method was introduced to increase the sample processing throughput using micro-flow LC [120,121,122,123]. As a result, a moderate loss of sensitivity, which was the key reason for using nano-flow LC, can result in improved robustness, throughput, and reproducibility. Recently, the same research groups proved the robustness of micro-flow LC and the potential for high-throughput clinical applications based on more than 38,000 proteomic samples collected over the past 2 years [124].

Despite the remarkable results of LC-MS-based diagnosis, further innovations in instruments and informatics are required for practical applications. The following section, therefore, focuses on informatics, which should be improved for future proteomics applications.

### 4.1. Data Deposition and Sharing Using Public Repositories

Data management has become important because of the rapid accumulation of MS data worldwide, produced by high-throughput MS (Figure 1C). However, enormous amounts of raw MS data can be useless if they are not quality controlled and well-organized using predefined terms, clinical metadata, and parameters used for analysis. Data repositories such as ProteomeXchange, Panorama, PeptideAtlas [125,126,127], and PRoteomics IDEntifications database (PRIDE) [128,129,130] are playing important roles in proteomics. The main characteristics or functionalities of each repository have been summarized in a recent paper [131]. Recently, most leading journals have mandated the deposition of raw data and analysis results in these public repositories [130]. Deposited data should be processed in a standard format that can be reused for further analysis. Accumulated datasets should be easily accessible and should be capable of being reanalyzed; this could be achieved by using an improved pipeline or other processes in a high-performance cloud computing environment [110]. These databases should also support other independent research results or benchmarking for new algorithms [110,132,133]. The integration of proteomic datasets with other omics datasets would expand the scope of our understanding of infectious diseases.

### 4.2. Expanding Community-Level Spectral Libraries

Similar to that with next-generation sequencing, it remains challenging to identify differentially abundant proteins, especially in the cases of proteins that occur at low abundance levels. Moreover, the larger the number of samples, the greater the number of missing values in the proteomics results, which will inhibit downstream analysis. To deal with this problem, missing values are currently replaced with reasonable values using various imputation methods, and statistical methods of transcriptomics are adopted. However, this can result in over-confident predictions [132,134,135,136]. Recently, intensity-dependent probabilistic modeling without imputation has been proposed to overcome this limitation [137].

However, the best answer for low-abundance proteins would be to increase proteome coverage to a reliable level. For this purpose, from an informatics perspective, spectral library approaches or hybrid searches could be applied to increase the proteomics depth; these approaches have been successfully used in DDA experiments [33,89,138,139,140,141]. Recent results have shown that search strategies can be critical for reproducibility, regardless of the acquisition method (DDA or DIA). This shows the importance of the spectral library approach [88,142]. However, the quality of spectral libraries from individual experiments is questionable, and the lack of publicly available libraries makes it difficult to apply spectral library search strategies to common proteomic data analysis [81,88,89,143,144,145,146]. For this reason, several synthetic peptide libraries, library-free approaches, or spectral prediction approaches have been developed as alternatives [147,148,149,150,151,152]. Although further improvements on deep learning-based methods have been achieved, these in silico approaches cannot completely replace experimentally derived libraries [150,153]. In addition, one of the major hindrances of this approach is that the series of library generation workflows is rather complicated, making it difficult for researchers to use. MaxDIA has recently enabled library-based and library-free DIA proteomics in the MaxQuant environment, making these approaches more intuitive for researchers to use [62]. Therefore, improving and accumulating public spectral libraries will aid in the development of the next generation of proteomics.

## Figures and Tables

**Table 1:** Characteristics of body fluids.

	Sample Type	Blood	BALF ^a^	CSF ^b^	Urine	Saliva	Ref.
Characteristics							
Non-invasive collection		Moderate	Moderate	No	Yes	Yes	[13]
Expert required		Moderate	Yes (local anesthesia)	Yes (local anesthesia)	No	No	[14]
Protein concentration (mg/mL)		60–80	0.05–0.2	0.2–0.8	0.08 ^c^	0.5–2	[14,15,16,17]
Complexity		Highest	High	High	Moderate	High	[18]
Proposed * SOPs for collection		Yes	Yes	Yes	Yes	Yes	[14,19,20,21,22]

* SOPs, standard operating procedures; BALF ^a^, bronchoalveolar lavage fluid; CSF ^b^, cerebrospinal fluid; ^c^ variable depends on hydration.

**Table 2:** Considered factors for protein sample preparation.

Factors	Description	Ref.
Immunodepletion	Immunodepletion is generally applied to remove high-abundance proteins and enrich low-abundance proteins.	[37,38,39,40]
Solubility of target proteins	MS-grade detergent can be applied to target low-abundance and hydrophobic proteins, such as membrane proteins.	[41]
Efficiency of protein preparation	The applicability of automation of protein isolation methods or extraction efficiency is critical to large-scale projects.	[42,43,44]
Peptide prefractionation or enrichment	Enrichment methods based on affinity binding require large starting protein amounts.	[18,45,46]

**Table 3:** Characteristics of LC-MS acquisition method.

	Acquisition Methods	DDA ^a^	MRM/PRM ^b^	DIA ^c^	Ref.
Characteristics					
Requirement for high-quality instruments		High	Moderate/High	Highest	[57]
Accuracy of protein quantification		Low	Highest	High	[58,59]
Reproducibility between replicates		Low	Highest	High	[59,60]
Depth of protein identification		Highest	Low	High	[58,59]
Ease of data analysis		Easy	Moderate	Hard	[61,62,63,64]

DDA ^a^, data-dependent acquisition; MRM ^b^, multiple reaction monitoring; PRM, parallel reaction monitoring; DIA ^c^, data-independent acquisition.

**Table 4:** Summary of body fluid proteomics for targeting pathogen-derived proteins.

Body Fluid	Study Groups	Sample Size	Target Pathogen	Major Findings	Method	Instrument	Ref.
Urine	Active pulmonary tuberculosis (TB)	9, 21	*Mycobacterium tuberculosis*	Four mycobacterial proteins were identified from the urine of nine patients. One of the candidate proteins was reconfirmed in urine from 21 clinical samples.	DDA	LCQ	[29,30]
Urine	Active TB vs. latent TB vs. non-TB	21 vs. 24 vs. 18	*Mycobacterium tuberculosis*	Ten mycobacterial proteins of active TB and six mycobacterial proteins of latent TB were identified.	DDA	LTQ-Orbitrap Velos Pro	[31]
CSF, Urine, Serum, and Saliva	Sleeping sickness early-stage vs. late-stage vs. uninfected	3 vs. 4 vs. 3	*Trypanosoma brucei gambiense*	Parasite proteins were identified but not further analyzed because of a lack of validity.	DDA	Q Exactive	[65]
Urine	Syphilis patient vs. Healthy	54 vs. 6	*Treponema pallidum*	The 26 unique peptides derived from 4 unique *T. pallidum* proteins were identified. These proteins have low sequence similarity to the human protein.	DDA, DIA	Synapt MS	[32]
Blood	Malaria patient	7	*Plasmodium vivax*	Five parasite-derived proteins of *P. vivax* were identified in 80% of patients.	DDA	6550 iFunnel Q-TOF	[66]
Urine	Urinary tract infection (UTI) patient	27	15 bacterial species ^a^	Eighty-two peptides were selected using machine learning classification and used for finding predominant pathogens from UTI patients.	DIA, PRM	Orbitrap Fusion, Q Exactive HF-X	[33]
Serum	pulmonary TB vs. extrapulmonary TB vs. latent TB vs. non-TB	31 vs. 10 vs. 9 vs. 9, 40	*Mycobacterium tuberculosis*	Twenty mycobacterial proteins were identified in the serum exosome of TB patients. The MRM assay can detect targets in the range of attomolar to femtomolar combined with isotope labeling.	MRM	Xevo TQ-S, LTQ-Orbitrap Velos	[67,68,69]
Nasopharyngeal and nasal swab	Respiratory tract infections patients	218	4 respiratory tract infection (RTI)-related bacterial species ^b^	Top 16–18 peptide biomarker candidates were selected for each of the four pathogens and verified using clinical samples.	PRM	Q Exactive, Q Exactive HF	[70,71]
BALF	Pneumonia patients	1	5 RTI-related bacterial species ^c^	Five unique peptides for each pathogen were selected according to abundance and applied for direct detection of pathogens.	MRM	Q-Exactive, Xevo TQ-S	[72]
endotracheal aspirate	VAP patients	37	6 RTI-related bacterial species ^d^	Ninety-seven species-specific peptides of the six pathogens, selected based on the proteotypicity and high ionization yield, were monitored and verified in clinical samples. The targeted proteomics assay showed 76% sensitivity and 100% specificity.	MRM	TripleTOF^®^5600 MS	[73]
nasopharyngeal swab	COVID-19 patient	9	SARS-CoV-2	To develop an assay, nasopharyngeal swabs with different quantities of viral material were used. The two peptides of N protein were selected. They can be obtained within 3 min of elution.	DDA	Q Exactive HF	[74,75]
nasopharyngeal swab	COVID-19 patient	103	SARS-CoV-2	The two peptides of the S protein were selected and monitored. The targeted assay showed 90.5% sensitivity and 100% specificity in a 2-min gradient run.	MRM	TripleTOF 6600	[76]
nasopharyngeal swab	COVID-19 patient	985	SARS-CoV-2	Fully automated sample preparation (SP3) and sample-cleanup methods (turbulent flow) were applied. The two peptides of the N protein were validated in a qualitative (Tier 3) and quantitative (Tier 1) manner. The targeted assay showed 84% sensitivity and 97% specificity in a 2.5-min gradient run.	PRM	Q Exactive HF-X	[77]
nasopharyngeal swab	COVID-19 patient vs. Healthy	88 vs. 88	SARS-CoV-2	Automated immunoaffinity-based sampling was applied. The two peptides of the N protein were selected for the targeted assay. The targeted assay was qualified using the ensemble method and showed 98% sensitivity and 100% specificity in a 5-min gradient run.	PRM	Orbitrap Exploris 480	[78]

^a^ *Citrobacter freundii*, *Enterobacter cloacae*, *Escherichia coli*, *Klebsiella aerogenes*, *Klebsiella oxytoca*, *Klebsiella pneumoniae*, *Pseudomonas aeruginosa*, *Proteus mirabilis*, *Enterococcus faecalis*, *Streptococcus agalactiae*, *Staphylococcus aureus*, *Staphylococcus epidermidis*, *Staphylococcus haemolyticus*, *Streptococcus mitis*, and *Staphylococcus saprophyticus*, ^b^ *S. aureus*; *Moraxella catarrhalis*; *Haemophilus influenzae* and *Streptococcus pneumoniae*; ^c^ *Acinetobacter baumannii*, *M. catarrhalis*, *P. aeruginosa*, *Stenotrophomonas maltophilia*, and *K. pneumoniae*; ^d^ *A. baumannii*, *E. coli*, *H. influenzae*, *P. aeruginosa*, *S. aureus*, and *S. pneumoniae*.
